# The influence of comorbid oppositional defiant disorder on white matter microstructure in attention-deficit/hyperactivity disorder

**DOI:** 10.1007/s00787-015-0784-3

**Published:** 2015-10-27

**Authors:** Hanneke van Ewijk, Siri D. S. Noordermeer, Dirk J. Heslenfeld, Marjolein Luman, Catharina A. Hartman, Pieter J. Hoekstra, Stephen V. Faraone, Barbara Franke, Jan K. Buitelaar, J. Oosterlaan

**Affiliations:** Department of Clinical Neuropsychology, Faculty of Psychology and Education, VU University Amsterdam, van der Boechorststraat 1, 1081 BT Amsterdam, The Netherlands; Department of Cognitive Psychology, VU University Amsterdam, Amsterdam, The Netherlands; Department of Psychiatry, University Medical Center Groningen, University of Groningen, Groningen, The Netherlands; Departments of Psychiatry and of Neuroscience and Physiology, SUNY Upstate Medical University, Syracuse, USA; Departments of Human Genetics and Psychiatry, Radboud University Medical Centre, Donders Institute for Brain, Cognition and Behaviour, Nijmegen, The Netherlands; Department of Cognitive Neuroscience, Radboud University Medical Centre, Donders Institute for Brain, Cognition and Behaviour, Nijmegen, The Netherlands; Karakter, Child and Adolescent Psychiatry, University Center Nijmegen in Nijmegen, Nijmegen, The Netherlands

**Keywords:** ADHD, Oppositional defiant disorder, Antisocial behaviour, Comorbidity, White matter microstructure, Diffusion Tensor Imaging

## Abstract

Attention-deficit/hyperactivity disorder (ADHD) and oppositional defiant disorder (ODD) are highly comorbid disorders. ADHD has been associated with altered white matter (WM) microstructure, though the literature is inconsistent, which may be due to differences in the in- or exclusion of participants with comorbid ODD. WM abnormalities in ODD are still poorly understood, and it is unclear whether comorbid ODD in ADHD may have confounded the current ADHD literature. Diffusion Tensor Imaging (DTI) was used to compare fractional anisotropy (FA) and mean diffusivity (MD) between ADHD patients with (*n* = 42) and without (*n* = 117) comorbid ODD. All participants were between 8–25 years and groups did not differ in mean age or gender. Follow-up analyses were conducted to examine the role of antisocial behaviour (conduct problems) on FA and MD values in both groups. Comorbid ODD in ADHD was associated with lower FA in left frontotemporal WM, which appeared independent of ADHD symptoms. FA was negatively associated with antisocial behaviour in ADHD + ODD, but not in ADHD-only. Comorbid ODD is associated with WM abnormalities in individuals with ADHD, which appears to be independent of ADHD symptoms. Altered WM microstructure in comorbid ODD may play a role in inconsistencies in the current DTI literature in ADHD. Altered development of these tracts may contribute to social-emotional and cognitive problems in children with oppositional and antisocial behaviour.

## Introduction

Attention-deficit/hyperactivity disorder (ADHD) is one of the most frequently diagnosed childhood psychiatric disorders, characterised by symptoms of inattention and hyperactivity/impulsivity which lead to functional impairment in daily life [1]. One of the greatest challenges in ADHD research is the large heterogeneity in aetiology, neurobiological correlates, and clinical presentation of the disorder. While the exact sources of this heterogeneity are still largely unknown, the presence of comorbidities may play a large role. In the majority of cases, ADHD is associated with one or more comorbid disorders, with oppositional defiant disorder (ODD) being among the most frequently diagnosed comorbidities with a prevalence rate of ~60 % in children and adolescents with ADHD [2].

Neuroimaging studies have consistently implicated abnormalities in brain structure and functioning in ADHD [3, 4], including differences in the microstructural properties of white matter (WM) tracts, as measured by Diffusion Tensor Imaging (DTI) [5]. WM tracts play an important role in information transfer between brain regions, and abnormalities in the microstructure or integrity of these tracts can have important implications for the structural and functional connectivity of the brain, which could ultimately result in neurocognitive deficits or behavioural problems. Meta-analytic evidence points towards reduced fractional anisotropy (FA) in the right anterior corona radiata, right forceps minor, bilateral internal capsule, and left cerebellum [6], consistent with theories on frontostriatal-cerebellar brain abnormalities in ADHD [7]. More recent DTI studies in ADHD implicate more widespread WM alterations, also including tracts that connect regions involved in sensorimotor and higher level cognitive functioning [8, 9]. However, DTI findings in ADHD are still highly inconsistent in terms of the location and direction of findings, impeding generalizability and interpretability of WM abnormalities in the disorder [6].

Given the high comorbidity of ODD in individuals with ADHD, it is possible that the inconsistencies in the ADHD DTI literature are—at least partly—due to differences between studies in the in- or exclusion of subjects with comorbid ODD. ODD is associated with widespread volumetric and functional brain abnormalities in regions including the frontal cortex, amygdala, and insula [10, 11]. However, to our current knowledge, no DTI studies of ODD have been performed, and it is currently unknown whether ODD is associated with differences in WM microstructure. DTI studies in conduct disorder (CD) have shown altered WM microstructure compared to controls in frontotemporal and striatal brain regions, represented by either lower [12] or higher [13, 14] FA values. Other studies reported lower FA and higher MD values in combined ODD/CD groups [15, 16], compared to controls. Although ODD and CD are distinct psychiatric disorders, they overlap substantially in terms of aetiology and pathophysiology, and ODD is often viewed as a milder variant of, or risk factor for, CD [17]. Consequently, based on the literature on WM microstructure in CD, it could be expected that WM abnormalities may also—at least to some extent—be implicated in ODD. One study directly compared individuals with ODD/CD + ADHD with those with ODD/CD without comorbid ADHD, and found that the comorbid group showed additional abnormalities in the corpus callosum and anterior, superior and posterior corona radiata [16]. These findings suggest that ADHD comorbidity in ODD/CD is associated with greater white matter abnormality than ODD/CD alone, and that comorbidity is an important factor to consider when investigating neurobiological correlates of these disorders.

Taken together, ADHD is associated with altered WM microstructure throughout the brain. WM abnormalities in ODD are still poorly understood, but may be expected in partly similar regions. Importantly, most previous DTI studies in ADHD have either not described the in- or exclusion of ODD, or included participants with ODD but did not test or describe the possible confounding effects. Therefore, it is possible that ODD-related WM abnormalities may have influenced the current DTI literature in ADHD, and that some of the WM abnormalities that are currently being attributed to ADHD may be better explained by the presence of comorbid ODD. To explore WM abnormalities in ODD, and shed light on the possible confounding effect of ODD-related abnormalities in ADHD DTI research, we compared WM microstructure between ADHD patients with and without comorbid ODD. We hypothesized that comorbid ODD would be associated with additional, or partly different, WM abnormalities compared to our previously reported ADHD-related WM abnormalities [5]. More specifically, we expected to find differences in FA and MD values between individuals with ADHD + ODD and ADHD alone, mainly located in frontotemporal and striatal WM, similar to previous findings in CD. To further elucidate the nature of WM microstructure abnormalities in ODD, we explored whether a dimensional measure of antisocial behaviour (conduct problems) was associated with WM abnormalities in both groups.

## Methods

### Participants

Participants were part of the NeuroIMAGE cohort [18]. Inclusion criteria for the current study were: age between 8 and 30 years, European Caucasian descent, IQ ≥ 70, and no known neurological or genetic disorder. Comorbid psychiatric disorders reported by parents (such as Bipolar disorder, classical autism or Asperer’s syndrome) were excluded, except for ODD, CD and pervasive developmental disorder not otherwise specified (PDD-NOS), given their high co-occurrence in ADHD, to avoid creating an extremely pure and non-representative sample of youth with ADHD. Complete data (i.e. information on ADHD, ODD and antisocial behaviour, and a DTI scan) were available for 159 individuals with ADHD, aged 8–25 years. Two groups were created: a “comorbid” group with ADHD + ODD (*n* = 42, including 7 participants with ODD + CD) and an “ADHD-only” group (*n* = 117; no ODD or CD).

Of note, group differences between ADHD and controls in the NeuroIMAGE cohort have been described in detail in a previous publication [5], in which we found decreased FA and increased MD in ADHD in widespread regions, and will thus not be repeated here. All ADHD participants from our previous publication were included in the current analyses, except for 11 participants with missing information on ODD or antisocial measures.

### Measures and materials

To determine ADHD and ODD diagnoses, all participants were assessed with the Schedule for Affective Disorders and Schizophrenia for School-Age Children-Present and Lifetime Version (K-SADS-PL) [19]. Parents were interviewed about their children’s behaviour during the past 6 months, and additionally, all children aged 12 and above were interviewed themselves, separate from their parents. Interview scores on each item were combined into an overall summary rating per item, including all sources of information (answers from parent, child (if applicable), and the interviewer’s clinical impression). ODD diagnoses were determined based on the K-SADS-PL using DSM-IV criteria. For ADHD, interview scores were supplemented with Conners’ questionnaires [20, 21] using a comprehensive diagnostic algorithm, resulting in a diagnostic category as well as the total number of ADHD symptoms. For a full description of the diagnostic procedures for ADHD we refer to a previous publication [18].

The presence of antisocial behaviour (conduct problems) was assessed with the Observed Antisocial Behavior Questionnaire (OAB) [22]. The OAB is based on the Self-Report of antisocial behaviour [23] and covers 42 antisocial and delinquent behaviours such as stealing, cheating, fighting and threatening others. The OAB was filled out by all participants in the absence of their parents, and young children (<12 years) who had trouble reading or filling out the questionnaire themselves were assisted by a research assistant. Of note, one item in the questionnaire assesses smoking behaviour. Given the well-established association between nicotine dependence and WM microstructure [24, 25], we decided to exclude this item from the questionnaire for the current study. Hereby, we minimized the possibility that our measure of antisocial behaviour—and related WM abnormalities—would be driven by the physiological effects of nicotine on WM microstructure, rather than conduct problems. From the remaining 41 items, a total score was calculated from all behaviours that had occurred during the past 6 months. Antisocial behaviour and diagnostic group were moderately positively correlated towards higher rates of antisocial behaviour for ADHD + ODD compared to ADHD-only (*r* = 0.32, *p* < 0.001).

Full scale IQ was estimated by a two-subtest short form (Vocabulary and Block Design) of the Wechsler Intelligence Scale for Children-III (WISC-III) or Wechsler Adult Intelligence Scale III (WAIS-III; for participants ≥17 years), to be used for exclusion of participants with IQ < 70. Additional information was collected to assess autism spectrum symptoms (using the Children’s Social Behavior Questionnaire; CSBQ), comorbid internalizing disorders (using the K-SADS-PL sections for depression and anxiety disorders), history of ADHD medication use (yes or no), and socio-economic status.

### Procedure

The current study was part of a comprehensive assessment protocol [18], including a DTI scan. Data acquisition was carried out in The Netherlands, either at the VU University Amsterdam and VU University Medical Centre, or at the Radboud University Medical Center and Donders Centre for Cognitive Neuroimaging in Nijmegen. Before the DTI scan, all participants were familiarized with the scanning procedure using a mock scanner. Participants were asked to withhold the use of psychoactive medication for 48 h before measurement. Fourteen participants were not able to comply, resulting in nine participants with a 24-h washout and five participants using medication during assessment (equally distributed between the two groups; *p* = 0.46). The study was approved by the Dutch local medical ethics committees, and all participants signed informed consent (parents signed for participants under 12 years of age). Afterwards, participants received a reward of €50 and a copy of their MRI scan.

### Imaging acquisition and (pre-)processing

MRI scanning was carried out on either a 1.5 Tesla Sonata or a 1.5 Tesla Avanto MRI scanner (Siemens, Erlangen, Germany), using the same Siemens 8-channel head coil. Whole-brain, high-resolution T1-weighted anatomical images were acquired in the sagittal plane (MP-RAGE, 176 slices, acquisition matrix 256 × 256, voxel size 1 × 1 × 1 mm; TE/TR = 2.95/2730 ms, TI = 1000 ms, FA = 7°, GRAPPA-acceleration 2). Eddy current-compensated diffusion-weighted SE-EPI images were collected during one acquisition consisting of five volumes without directional weighting (*b* value of zero), followed by 60 volumes with non-collinear gradient directions (60 interleaved slices, matrix 64 × 64, voxel size 2 × 2 × 2.2 mm, TE/TR = 97/8500 ms, *b*-value 1000 s/mm^2^, GRAPPA-acceleration 2).

Pre-processing included eddy current correction, realignment (using affine transformations and mutual information as a cost function), unwarping image distortions, and correction of motion-induced artefacts, using SPM8 (Wellcome Trust Centre for Neuroimaging) functionality and in-house developed methods. Tensor images were estimated, and FA and MD maps were derived for each participant, which were further processed using FSL’s Tract-Based Spatial Statistics (TBSS) [26] using standard settings with a threshold of FA > 0.3. For a full description of image (pre-)processing we refer to our previous publication [5].

### Data analysis

Group differences in sample characteristics were investigated using analysis of variance and Chi square tests in SPSS (version 21, IBM, Chicago, IL, USA). Voxelwise TBSS analyses were performed in FSL with the randomise tool, which is based on permutation testing. A General Linear Model (GLM) was built with diagnostic group as predictor (ADHD + ODD versus ADHD-only). Covariates were included for the number of ADHD symptoms (given the group difference in ADHD symptoms), age (given our broad age range and the strong association between age and WM microstructure) and scan site (to correct for possible site differences). First, *t* test contrasts were set up to investigate main group differences (ADHD + ODD versus ADHD-only) on FA and MD. Second, additional contrasts were set up for group-by-age and group-by-ADHD symptom count interactions, to investigate whether group differences in FA or MD differed as a function of age or ADHD symptom count. Results were obtained using Threshold-Free Cluster Enhancement (TFCE), providing results at *p* < 0.05 using FWE correction for multiple testing. Anatomical labels of voxels that showed significant effects were identified using the built-in Harvard-Oxford and JHU atlases for WM tracts in FSLview. Given the group differences in IQ and autism spectrum symptoms (see Table 1), and the literature supporting strong associations of IQ and autistic traits with white matter microstructure [27, 28], we ran additional analyses with IQ and autism spectrum symptoms added to the GLM as covariates.

**Table 1:** Sample characteristics

	ADHD-only (*n* = 117)	Comorbid (*n* = 43)	Test statistics	*p* value
Age (*M*, SD)	17.28 (3.37)	17.13 (3.26)	*F*_1,157_ = 0.06	0.803
Gender (*N*, % male)	79 (68 %)	28 (67 %)	*χ*_1,*N*_^2^_=159_ = 0.53	0.919
Scan site (*N*, % Amsterdam)	46 (39 %)	20 (48 %)	*χ*_1,*N*_^2^_=159_ = 0.88	0.349
Number of ADHD symptoms (*M*, SD)	12.59 (2.83)	14.00 (2.96)	*F*_1,157_ = 7.50	0.007
Oppositional behaviour (*M*, SD)^a^	55.22 (10.42)	69.81 (11.92)	*F*_1,154_ = 55.62	<0.001
Antisocial behaviour (*M*, SD)^b^	3.57 (3.73)	6.21 (4.3)	*F*_1,157_ = 14.29	<0.001
Vocabulary standardized score (*M*, SD)	9.43 (2.66)	8.38 (2.52)	*F*_1,156_ = 4.93	0.028
IQ (*M*, SD)	100.03 (15.14)	92.85 (11.82)	*F*_1,157_ = 7.76	0.006
Hand preference (*N*, % right handed)	102 (88 %)	38 (91 %)	*χ*_1,*N*_^2^_=159_ = 0.47	0.792
Socio-economic status (*M*, SD)	11.65 (2.25)	11.20 (2.01)	*F*_1,155_ = 1.33	0.251
Internalizing disorder (*N*, %)	3 (3 %)	3 (7 %)	*χ*_1,*N*_^2^_=157_ = 1.85	0.174
Autism spectrum symptoms (*M*, SD)	9.93 (7.42)	16.07 (8.26)	*F*_1,154_ = 19.79	<0.001
History of ADHD medication use (*N*, %)	101 (89 %)	37 (90 %)	*χ*_1,*N*_^2^_=154_ = 0.02	0.877

^a^ *T*-score; as measured by the Conners Parent Rating Scale

^b^Standardized score

Subsequent follow-up analyses were conducted to further examine the nature of the significant group differences. All follow-up analyses were conducted in SPSS on mean FA or MD values from regions in which a significant group difference was found in the TBSS analysis. Linear Mixed Models (LMM) were used with a random intercept per family, to account for correlated data within families, and the same predictors and covariates were included as in the TBSS analysis (i.e. diagnostic group, ADHD symptoms, age and scan site). First, to check whether our results were applicable to all ages in our broad age range, we performed a median split on age and re-ran the LMM on both age groups separately. Second, to investigate whether lower FA in the ADHD + ODD group could be explained by higher rates of antisocial behaviour, the LMM was run with a dimensional measure of antisocial behaviour (normalized with van der Waerden’s transformation) and its interaction with diagnostic group as predictors. Last, to test whether participants with comorbid CD had confounded our main effect or the interaction with antisocial behaviour, both LMMs were re-run after excluding the participants with comorbid CD (*n* = 7).

## Results

### Sample characteristics are summarized in Table 1

**Fig. 1:**
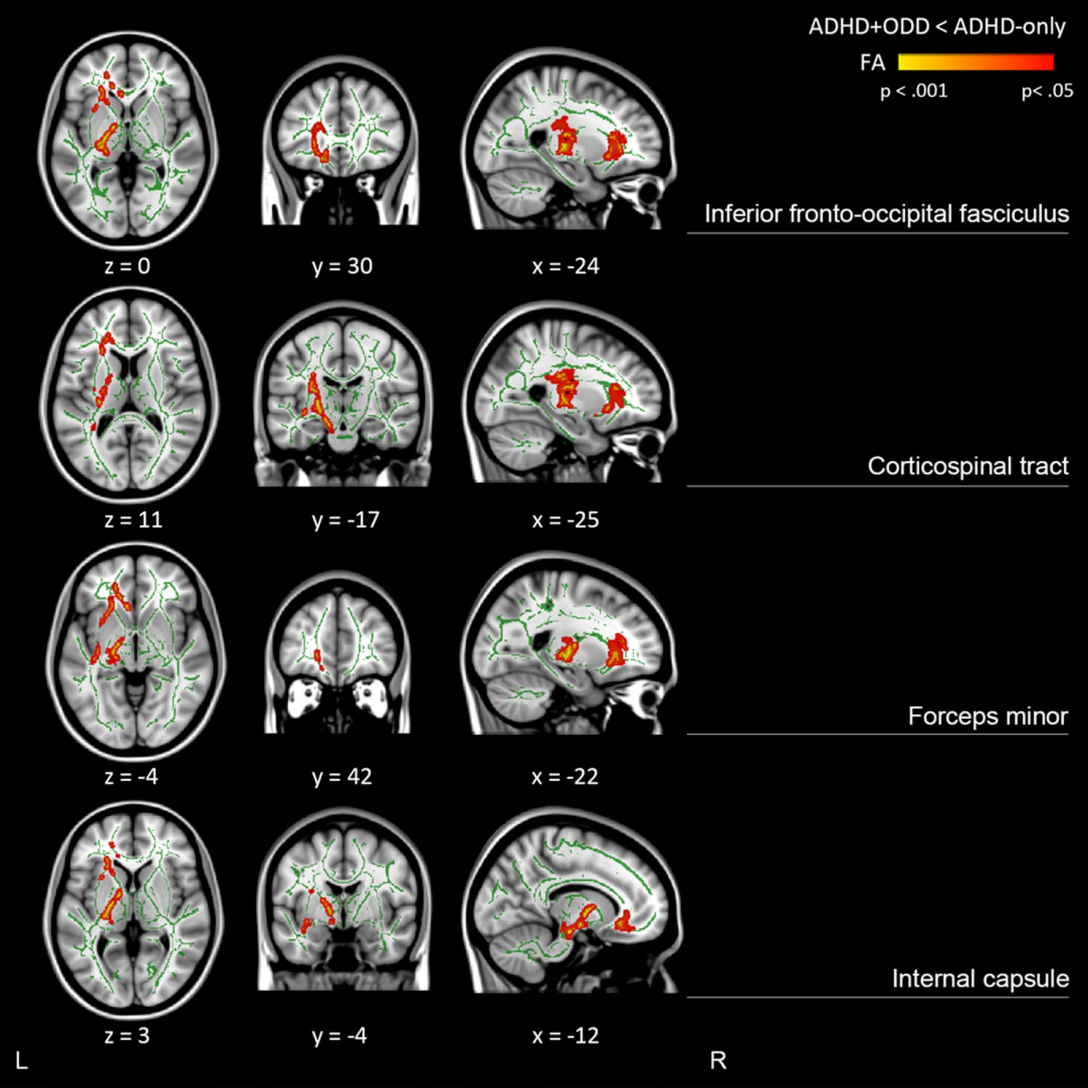
Tract-based spatial statistics (TBSS) results of fractional anisotropy (FA). *Red* and *yellow colours* represent regions of lower FA for ADHD + ODD compared to ADHD-only. Results are overlaid on a standard MNI152 template with the mean skeleton (*green colour*; FA > 0.3) and were “thickened” towards the full width of the tract for visualization purposes. Anatomical labels refer to major white matter tracts that are visible in the corresponding view, as identified with JHU white matter atlases incorporated in FSL

The TBSS analysis revealed several clusters of decreased FA for ADHD + ODD compared to ADHD-only (Fig. 1; Table 2). Clusters of decreased FA were mainly located in WM tracts and subcortical structures, more specifically in the inferior fronto-occipital fasciculus or uncinate fasciculus, corticospinal tract, anterior and posterior capsula interna, and forceps minor or genu of the corpus callosum. All findings were lateralized to the left hemisphere. For each participant, the mean FA value was extracted from these voxels for follow-up analyses. No differences in MD and no areas with increased FA for ADHD + ODD were found, and no interactions between group and age or ADHD symptom count were observed for either FA or MD. Re-running the GLM with IQ added to the GLM as a covariate revealed the same results, albeit somewhat more widespread. Newly unmasked voxels were mainly located in the bilateral forceps minor and right anterior corona radiata. Re-running the GLM with autism spectrum symptoms added to the GLM as a covariate revealed results very similar to the original analysis, although some voxels, located in the lower portion of the CST and the more medial part of the forceps minor, had disappeared just under the significance threshold of *p* < 0.05). Results from these additional analyses show that group differences in FA were robust for subclinical autism spectrum symptoms and are present at all levels of cognitive functioning.

**Table 2:** Clusters of lower fractional anisotropy (FA) for ADHD + ODD compared to ADHD-only

Cluster #	Anatomical label	*n* Voxels	MNI coordinates			*p*
			*x*	*y*	*z*	
1	Corticospinal tract, internal capsule (anterior and posterior), forceps minor/genu of corpus callosum	1300	−19	−12	−1	0.028
2	Inferior fronto-occipital fasciculus/uncinate fasciculus	1297	−14	29	−12	0.034
3	Inferior fronto-occipital fasciculus	125	−33	−58	20	0.046

MNI coordinates represent centre of gravity. Only clusters with cluster size >100 voxels are shown

**Fig. 2:**
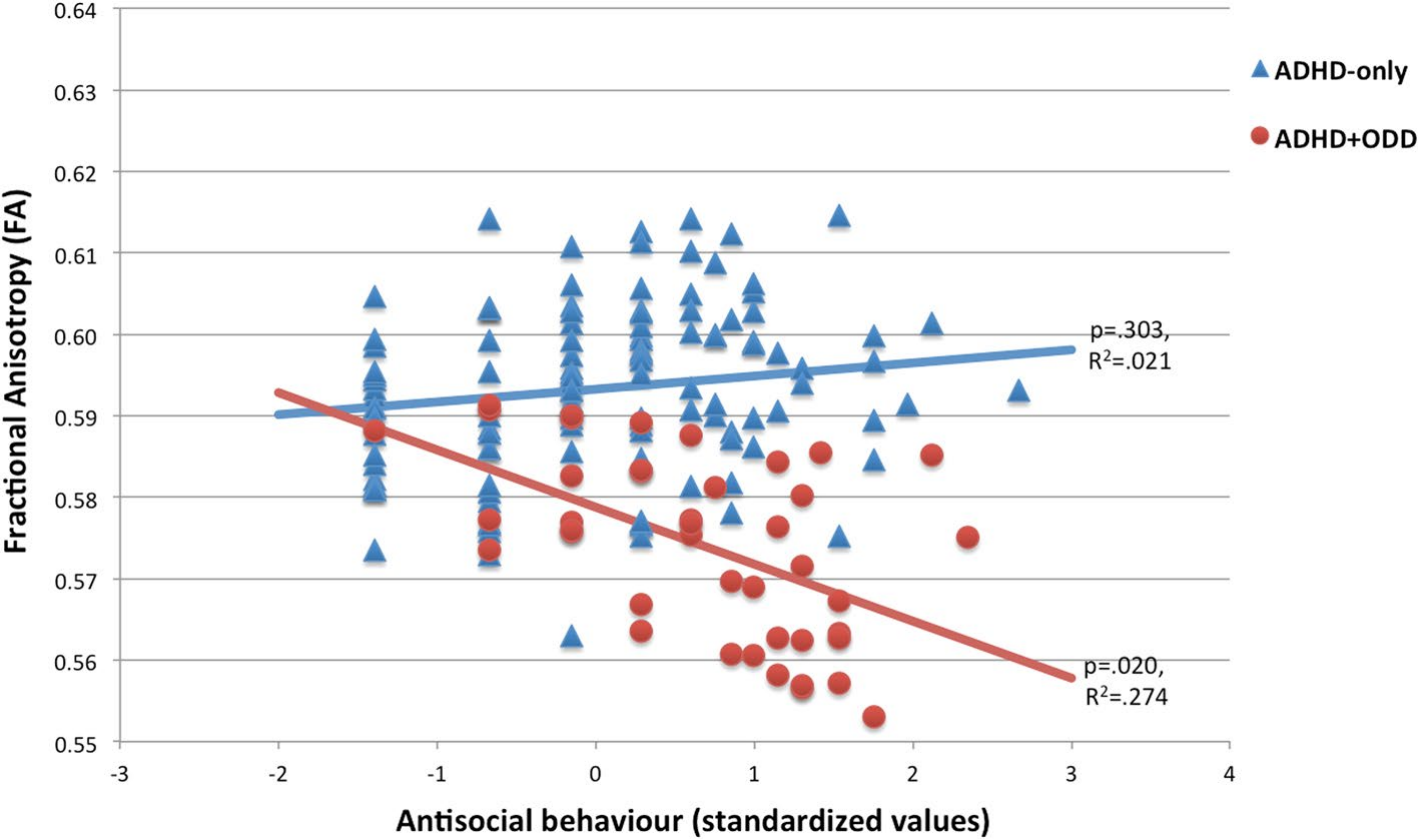
Interaction between antisocial behaviour (conduct problems) and group status on fractional anisotropy (FA). Mean FA values were extracted from clusters that showed a significant group difference (lower FA for ADHD + ODD versus ADHD-only) in the Tract-based spatial statistics (TBSS) analysis

Follow-up analyses were conducted in SPSS on mean FA values from the significant voxels of reduced FA in ADHD + ODD. First, we re-ran the LMM in two separate age groups, split at the median age (17.5 years). Results remained the same for both the younger and the older age group. Second, we examined the association between diagnostic group and antisocial behaviour as measured by the OAB (Fig. 2). A significant interaction was found (*F*_1,152_ = 6.658, *p* = 0.011), which was further examined by testing the association between antisocial behaviour and FA in each diagnostic group separately. Results revealed a significant negative association between antisocial behaviour and FA in the ADHD + ODD group (*F*_1,37_ = 5.939, *p* = 0.020) but not in ADHD-only (*F*_1,112_ = 1.073, *p* = 0.303). Excluding participants with comorbid CD (*n* = 7) did not change the significance of the main group effect or the interaction with antisocial behaviour. Additional follow-up analyses were conducted to examine whether the observed left-sided lateralization of TBSS findings was associated with language impairment in ODD. To this end, the LMM was re-run with the Vocabulary standardized score of the WISC-III/WAIS-III and its interaction with diagnostic group as predictors. Results revealed no effect of Vocabulary on FA (*F*_1,143_ = 0.607, *p* = 0.437) and no significant interaction with diagnostic group (*F*_1,147_ = 0.075, *p* = 0.784).

## Discussion

This is the first study investigating WM microstructure associated with comorbid ODD in ADHD. We aimed to shed light on WM microstructure in ODD as well as the possible confounding effect of ODD comorbidity on previous DTI findings for ADHD.

Several regions of reduced FA were found in individuals with ADHD + ODD compared to those with ADHD alone, mainly located in frontotemporal WM tracts and subcortical regions of the basal ganglia in the left hemisphere. More specifically, reduced FA was located in the left corticospinal tract, inferior fronto-occipital fasciculus or uncinate fasciculus, forceps minor or genu of the corpus callosum, and internal capsule. No differences in MD were observed between the groups, and group differences in FA were not dependent on ADHD symptom count. These findings could indicate an additive or differential effect of ODD on ADHD-related WM pathology, in line with a previous study that showed a similar pattern of findings for adolescents with ODD/CD with and without comorbid ADHD [16]. Of note, significant voxels were often located in regions in which two or more major white matter tracts are located or crossing (e.g., inferior fronto-occipital fasciculus and uncinate fasciculus). One of the disadvantages of a voxel-based approach like TBSS is that it is not possible to infer information regarding the specific tract that was measured, and FA values cannot be interpreted in terms of the biological properties underlying a difference in anisotropy, especially in regions of crossing fibres [29, 30].

Importantly, current results show some striking similarities and differences with results from our previous study in a largely overlapping sample, in which we found reduced FA for ADHD compared to controls in widespread regions [5]. Qualitative comparison between the two studies reveals WM abnormalities in left temporal and striatal regions for ADHD compared to controls, but also for ADHD + ODD compared to ADHD patients without comorbid ODD. This could indicate an additive effect of ADHD and ODD on FA in temporal and striatal regions, where individuals with ADHD + ODD show more severe WM abnormalities than those with ADHD alone. On the other hand, left (orbito)frontal regions show reduced FA for ADHD + ODD compared to ADHD-only, but not for ADHD compared to controls, indicating that these abnormalities may be more specific to ODD than ADHD. It is important to note that direct quantitative comparisons between both studies cannot be made due to slight differences in sample composition and statistical methods such as the included covariates. Described overlap and differences between both studies should be seen as exploratory, and interpreted with caution.

Our findings are helpful in resolving the inconsistencies in the prior DTI literature for ADHD by showing that ADHD patients with and without ODD differ in WM microstructure in several tracts. It is possible that findings from previous studies, especially in (orbito)frontal WM regions, have been attributed to ADHD, while they may better be explained by comorbid ODD in the sample. Furthermore, individuals with comorbid ODD appear to have more severe WM abnormalities than individuals with ADHD alone in temporal and striatal regions, which may have led to differences in the strength of effects in previous studies that differ in their in- or exclusion strategies. Future studies should clearly test and describe the possible confounding effect of ODD comorbidity in their sample, and current findings should be kept in mind in interpreting previous literature in which ODD comorbidity was not described or excluded.

Antisocial behaviour, or conduct problems, interacted with group status in such a way that WM abnormalities were mainly present in individuals with comorbid ODD *in combination with* high rates of antisocial behaviour. Comorbid ODD with low rates of antisocial behaviour, and antisocial behaviour without an ODD diagnosis, did not appear to be associated with lower FA. Importantly, our measure of antisocial behaviour largely overlapped with CD-like behaviours. Therefore, it is possible that our finding in fact signifies an interaction between ODD and (subclinical) CD, and that WM pathology is strongest in individuals with both disorders combined. Although ODD and CD are highly correlated constructs, this result suggests that both disorders may interact on a neurobiological level, indicating that ODD and CD should be treated as separate constructs in future studies investigating WM microstructure. It is important to note that young children in our sample may still go on to develop ODD and/or CD in the future. Hence, it is difficult to know whether the currently defined subgroups will hold in the future, or whether children may shift from the ADHD-only to the Comorbid group in a few years’ time, and if so, whether group differences in WM microstructure will remain the same. It would be informative to replicate current results in a longitudinal sample following ODD and conduct symptoms, as well as WM microstructure, over time.

Current findings fit well within theories of frontotemporal and frontostriatal brain dysfunction in individuals with ODD and/or CD [31–33]. The basal ganglia are well known for their central role in the reward circuitry and emotional functioning, and the orbitofrontal cortex plays a crucial role in controlling representational memory, incentive motivation, and reward processes. These are all well-known cognitive difficulties in ODD and CD [17], and reduced brain activation in these regions has consistently been linked to aggression and psychopathy [34]. The uncinate fasciculus, connecting the orbitofrontal cortex with temporal lobe regions, plays an essential role in combining reward and punishment history, memory representations, value assignment and updating, and decision making [35]. As a consequence, perturbation of this tract can cause problems in social-emotional functioning, due to the lack of emotional history and value and motivational value in the decision-making process. It is likely that WM abnormalities in frontotemporal and frontostriatal brain regions play a role in the neurocognitive and behavioural problems associated with ODD, as well as the poor and adverse outcomes reported for children with ADHD + ODD compared to those with ADHD alone [2, 36]. At the neurobiological level, reduced FA could implicate abnormalities in a wide range of tissue properties such as reduced myelin or lower axonal density [29], which could signify disrupted signal transfer in these tracts. Importantly, WM microstructure in frontotemporal and striatal regions has been shown to continue to develop into adulthood in healthy subjects, with increasing FA and decreasing MD over age [37]. Given that our sample largely consisted of adolescents, it is unclear whether our finding of reduced FA in comorbid ODD represents a developmental delay (compared to individuals with ADHD alone), which could catch up in adulthood, or whether it signifies a more persistent deficit. Longitudinal studies could provide more insight. Taken together, lower FA in comorbid ODD could represent suboptimal development of frontotemporal WM tracts, which could play a role in the social-emotional and cognitive problems associated with ODD.

Tracts of lower FA in comorbid ODD were lateralized to the left hemisphere, suggesting that findings may be related to a lateralized brain function such as handedness or language functioning. While handedness is unlikely to explain the lateralization of our results, given that our groups did not differ on handedness, our groups did differ on vocabulary skills, and language deficits have been suggested to play a role in disruptive behaviour disorders such as ODD [38]. DTI studies have shown that WM microstructure of the genu [39] and the splenium [40] of the corpus callosum and the left inferior fronto-occipital fasciculus [41] is associated with language abilities and impairment. Functional magnetic resonance imaging (fMRI) evidence also suggests a role of the uncinate fasciculus in language processing [42], although evidence for this tract is less consistent. It is possible that suboptimal development of frontotemporal and callosal tracts may signify a shared pathophysiology underlying both the language difficulties and behavioural symptoms of ODD. This theory was not directly supported by our data, in which FA was not associated with vocabulary scores. However, disruptive behaviour disorders are associated with a wider range of language difficulties, including receptive listening and reading and expressive speech and writing, which were not explicitly assessed in the current study. Therefore, other indices of language and reading ability than vocabulary may in fact be associated with WM microstructure in these regions.

Our current findings should be viewed in the light of some strengths and limitations. Strengths include our large sample size, 60-direction diffusion imaging, and the possibility to check for a wide variety of confounding factors, which was a caveat in many studies performed so far. The lack of a pure ODD group restricts the generalizability of findings to comorbid ODD in ADHD, but not to pure ODD (without ADHD). Nevertheless, ODD did not interact with ADHD symptom severity, which suggests that our findings for comorbid ODD appear to be largely independent of ADHD and may generalize to pure ODD. This is especially the case for findings in (orbito)frontal regions, which we did not find in our previous ADHD-control comparison [5]. However, a study combining four groups (controls, pure ODD, pure ADHD, and a comorbid group) could shed more light on the specificity of WM abnormalities for both disorders. The age range in the current study was relatively broad, restricting us from drawing conclusions regarding specific age groups. Although our main TBSS finding appeared to be robust for age, it is possible that additional WM tracts may be involved specifically in younger or older individuals, which should be explored in a sample with a more specific age group with an appropriate sample size. Due to the cross-sectional nature of the current study, it is not possible to draw conclusions regarding causality of the findings, i.e. whether WM abnormalities cause ADHD and ODD behaviours and associated neurocognitive deficits, or vice versa. Longitudinal studies could provide more insight into the development of WM microstructure and associated functions throughout the childhood and adolescence in individuals with and without ODD.

## Conclusion

The current study adds significantly to the scarce literature on WM microstructure in ODD and is the first to describe the effects of comorbid ODD in ADHD. Our results show that comorbid ODD in ADHD is associated with altered WM microstructure, compared to individuals with ADHD alone, suggesting more severe and partly different WM abnormalities in comorbid individuals. WM pathology was strongest in individuals with comorbid ODD in combination with high rates of antisocial behaviour (conduct problems) and was mainly located in frontotemporal and striatal regions. It is possible that altered development of these tracts during childhood may underlie problems with social-emotional decision making, reward processing and motivational control, and predispose to the development of oppositional and antisocial behaviour, although longitudinal studies are needed to investigate the causality of this association.
